# Pertussis outbreak mainly in unvaccinated young children in ultra-orthodox Jewish groups, Jerusalem, Israel 2023

**DOI:** 10.1017/S0950268823001577

**Published:** 2023-09-22

**Authors:** Chen Stein-Zamir, Hanna Shoob, Nitza Abramson, Emilie Hannah Brown, Yael Zimmermann

**Affiliations:** 1 Jerusalem District Health Office, Ministry of Health, Jerusalem, Israel; 2 The Hebrew University and Hadassah Braun School of Public Health and Community Medicine, The Hebrew University of Jerusalem, Faculty of Medicine, Jerusalem, Israel; 3 School of Public Health, University of Miami, Miami, FL, USA

**Keywords:** *Bordetella pertussis*, outbreak, pertussis (whooping; cough), ultra-orthodox, vaccines

## Abstract

Despite being a vaccine-preventable disease for decades, pertussis control is still a public health challenge. A pertussis outbreak emerged in Jerusalem (*n* = 257 cases, January to June 2023). Most cases were young children (median age 1.5 years), and 100 were infants under 1 year. The hospitalisation rate of infants was 24%, which was considerably higher than that of cases aged 1 year and above (3.8%). There was one fatality in an unvaccinated, 10-week-old infant whose mother had not received pertussis vaccination during pregnancy. Most children were unvaccinated and resided in Jewish ultra-orthodox neighbourhoods in Jerusalem district. An intervention programme and vaccination campaign are ongoing.

## Introduction

*Bordetella pertussis* infection is transmitted from person to person via exposure to the respiratory secretions of infected individuals, primarily through large droplets produced during coughing or sneezing [1]. In epidemiological studies, the highest disease burden of pertussis (including the age-specific incidence rates, pertussis hospitalisations, complications, and fatalities) has been observed in infants under 1 year [2–4]. The peak pertussis burden occurred in infants under 6 months, who were not yet eligible for the full pertussis vaccination schedule or unvaccinated [4]. In a modelling study on the global burden of pertussis among young children (for 2014), an estimated 24.1 million pertussis cases and 160,700 deaths occurred among children under 5 years of age, with an estimated 5.1 million (21%) pertussis cases and 85,900 deaths (53%) among infants under 1 year of age [5].

Despite being a vaccine-preventable disease for decades, pertussis has remained a global public health challenge. Resurgence of pertussis in the vaccination era has been reported, even in countries with well-established routine childhood vaccination programmes [6]. In Israel, the current routine childhood immunisation programme includes the pentavalent diphtheria–tetanus–acellular pertussis–polio–*Haemophilus influenzae* type b vaccine (DTaP-IPV-Hib) scheduled at the ages of 2 months, 4 months, 6 months, and 12 months [7]. In schoolchildren, the tetanus–diphtheria–acellular pertussis vaccine (Tdap) is recommended at ages 7 years and 13 years [7]. Pertussis vaccinations during pregnancy were included in the national health basket in Israel in 2015 [7]. A single Tdap dose is recommended for pregnant women in the third trimester (in weeks 27–36) of each pregnancy; vaccinations in pregnancy are provided by the health funds [7]. Routine childhood vaccinations in Israel are free of charge and provided to children in community-based well-baby clinics (for ages birth – 6 years) and by school health services (for ages 6–15 years); all vaccination data are digitally documented in computerised health records and incorporated into the national immunisation registry [8].

Overall, the rates of respiratory infections showed a trend of decline during the years of the global COVID-19 pandemic (2020–2022), probably associated with reduced transmission with the implementation of containment directives such as lockdowns, various social distancing measures, and extensive utilisation of face masks [9, 10]. In 2023, an increase in respiratory infection rates (including pertussis) have been noted in several countries, and pertussis outbreaks emerged and were reported from Canada, the Philippines, South Africa, Bolivia, the United States of America, Malaysia, and Israel [11]. In early 2023, an increase in the number of pertussis notifications have been observed in Israel, with most pertussis cases reported in Jerusalem district. We aimed to describe the epidemiology of the pertussis outbreak in Jerusalem district during January–June 2023.

## Outbreak description

A rise in pertussis notifications in Jerusalem district has been noticed in January 2023. The number of pertussis notifications in the district during January–June 2023 was 257 compared to 19 notified pertussis cases in 2022 and four cases in 2021. Most pertussis cases notified nationally during January–February 2023 (94.9%, 56/59) were reported in Jerusalem district. The number of notified pertussis cases from other districts increased gradually during March–June 2023, with Jerusalem district comprising 63.7% (201/314) of the cases nationally.

*B. pertussis* infection is legally notifiable in Israel; physicians and laboratories notify cases to the district health office in charge of the epidemiological investigations and infection control measures [12]. The epidemiological data collected include age, gender, address, date of disease onset, laboratory tests, hospitalisation, and outcome and vaccination status. Additional variables collected in cases of infants under 1 year include birthweight, birth order, mother’s age, and receipt of pertussis vaccine during pregnancy. The pertussis laboratory confirmation is predominantly based on polymerase chain reaction (PCR) tests. Data analysis is performed with Statistical Package for Social Sciences, SPSS® software. Variables are compared by the Student *t* test, Pearson chi-square test, and odds ratio (OR) with 95% confidence interval (95%CI), as appropriate. A p-value <0.05 is considered significant for all comparisons.

The population of Jerusalem district is 1.37 million (2022, 14.6% of Israel’s general population). Children under 5 years (178,000) and infants under one year (37,000) comprise 13% and 2.7% of the district’s population, respectively. Jerusalem district livebirths are recorded in the district’s newborn registry. Notably, Jerusalem district’s infants comprise approximately 20% of the national cohort (185,000, 2022), attributable to the district’s population high fertility rates.

The general characteristics of the notified pertussis cases in Jerusalem district during January–June 2023 (*n* = 257) are presented in Table 1. The vast majority of the pertussis cases were children, 92.2% of them being under 19 years of age. The median age of the outbreak’s pertussis cases was 1.5 years. Infants under 1 year of age consisted the largest group (*n* = 100). In the pertussis cases in infants under 1 year, the median age was 5 months. The largest fraction has been observed in infants aged 0 to 3 months (*n* = 35 cases). Most of the infants were born with a normal birthweight, and 7% had a birthweight less than 2500 grams. The pertussis cases in infants had a median birth order of five (mean 5.4 ± 3.3), and approximately 67% of them had a birth order of four and above. According to Jerusalem district newborn registry, the overall median birth order was three (mean 3.5 ± 2.4, 2022 cohort). The pertussis cases in infants showed a significantly higher mean birth order than the district cohort (*P* < 0.001).

**Table 1.** General characteristics of the pertussis cases, Jerusalem district, January–June 2023

Variable	Cases (*n* = 257)
Age (years) mean ± SD	6.8 ± 12.5
Age (years) median (interquartile range)	1.5 (0.6–7.7)
Gender, male (%)	115 (44.7%)
Age group	
0–3 months	35 (13.6%)
4–6 months	18 (7%)
>6 months–1 year	47 (18.3%)
1–6 years	85 (33.1%)
7–18 years	52 (20.2%)
19 years and above	20 (7.8%)
Hospitalization (all cases)	30 (11.7%)
Hospitalization infants under 1 year	24 (24%)
Hospitalization persons 1 year and above	6 (3.8%)
Data for infants under 1 year (*n* = 100)	
Birthweight (g) mean ± SD	3,284 ± 553
Birthweight (median) (interquartile range)	3,335 (3,053–3,655)
Birth order (mean ± SD	5.4 ± 3.3
Birth order (median) (interquartile range)	5 (3–7)
Mother’s age (years) mean ± SD	30.9 ± 7.1
Mother’s age (years) median (interquartile range)	31 (25–37)
Mother’s birth country Israel	89 (89%)

The hospitalisation rate observed among infants under 1 year of age (24%) was significantly higher than that in persons aged 1 year and above (3.8%) (OR = 7.9, 95%CI 2.9–24.6, *P* < 0.001). The hospitalisation rate also differed significantly between pertussis cases in infants under 3 months (54.3%) and those aged 3–12 months (7.7%) (*P* < 0.001).

Overall, infants under 1 year accounted for most pertussis-related hospitalisations (80%) in Jerusalem district during January–June 2023 and one death. The fatal case occurred in a previously healthy, unvaccinated, 10-week-old infant whose mother had not received pertussis vaccination during pregnancy. The infant’s clinical symptoms included coughing fits, whooping, vomiting, apnoea and cyanosis. Pertussis PCR was positive. The hospitalisation occurred at age 7 weeks with need for mechanical ventilation and later extracorporeal membrane oxygenation (ECMO); after 3 weeks of hospitalisation, the infant succumbed to multi-organ failure.

The vaccination status of each case has been confirmed against the national vaccination registry, in the children aged 0–18 years and based on the epidemiological investigations in persons aged 19 years and above. The vaccination status of the pertussis cases (according to age groups), presented as the distribution of the number of prior pertussis vaccine doses, is shown in Table 2. Of the outbreak’s pertussis cases, about 30% had received at least one pertussis vaccine dose, and around 70% were unvaccinated. The vaccinated fraction among infants aged 2 months to 1 year (eligible for the first pertussis vaccine dose, *n* = 80) was 15% (12/80), and in children aged 1 to 2 years, the vaccinated fraction was 14.3% (6/42). All the pertussis cases in children aged 0–6 years were evaluated with all the preventive health service providers in Jerusalem district; none of the unvaccinated children had been registered in any of the district’s well-baby clinics.

**Table 2.** Vaccination status (number of doses of pertussis vaccine) of the pertussis cases, Jerusalem district, January–June 2023

Age group	*n*	Unvaccinated (%)	1 dose	2 doses	3 doses	≥4 doses
All cases	257	177 (68.9%)	17 (6.6%)	1(0.4%)	9 (3.5%)	53 (20.6%)
0–3 months	35	29 (82.9%)	6 (17.1%)	0	0	0
4–6 months	18	16 (88.9%)	1(5.6%)	0	1(5.6%)	0
>6 months–1 year	47	43 (91.5%)	1 (2.1%)	0	3 (6.4%)	0
Total < 1 year	100	88 (88%)	8 (8%)	0	4 (4%)	0
1–6 years	85	67 (78.8%)	8 (9.4%)	0	2 (2.3%)	8 (9.4%)
7–18 years	52	9 (17.3%)	1 (1.9%)	1 (1.9%)	3 (5.8%)	38 (73.1%)
19 + years	20	13 (65%)	0	0	0	7 (35%)

In the group of children aged 7–18 years (*n* = 52, mean age 11 ± 2.9 years, median 11 years), 38 (73.1%) had received four or more pertussis vaccine doses. The mean time between the last vaccine dose and the date of reported pertussis diagnosis was 5 ± 2.7 years, median 5.4 years.

Regarding receipt of pertussis vaccination during pregnancy in mothers of infants’ cases, 84 of 100 mothers had not received vaccination. Sixteen mothers reported receiving pertussis vaccination in the third trimester of pregnancy. In the vaccinated mothers, the mean number of days between the date of pertussis vaccine receipt and the childbirth date was 65 ± 26.8 days (median 71.5 days). Three of the vaccinated mothers received the pertussis vaccine 14 days or less before childbirth. The mother and infant vaccination status were positively associated. Among the infants aged 2 months to 1 year – 53.3% (8/15) of infants, whose mothers were vaccinated in pregnancy received one pertussis vaccine dose compared to 6.1% (4/65) of infants whose mothers were not vaccinated during pregnancy (OR = 17.4, 95%CI 3.4–95.6, *P* < 0.001).

The notification of additional pertussis cases in the same household has been observed in 33 of 257 (12.8%) of the outbreak cases. Two pertussis cases have been notified in thirteen households, and three and four pertussis cases have been notified in one household each.

The 2023 pertussis cases were evaluated according to the cases’ geographic areas of residency in Jerusalem district, in search for clusters. Most pertussis cases resided in the ultra-orthodox Jewish neighbourhoods in the city of Jerusalem (108/275, 42%), the ultra-orthodox Jewish neighbourhoods of Beit Shemesh (BS, 94/275, 36.6%), and the ultra-orthodox Jewish town Beitar Illit (BI, 25/257, 9.7%) in communities that are highly interconnected. There were certain streets with large numbers of pertussis cases; 23 cases were reported in one street in an ultra-orthodox neighbourhood in BS, and 17 cases in one street in a neighbourhood in an ultra-orthodox neighbourhood in central Jerusalem. Notably, the spread of pertussis to other districts in the country (mainly reported in unvaccinated children) has been epidemiologically linked to the cases in Jerusalem district, through various familial and social interactions.

**Figure 1.**
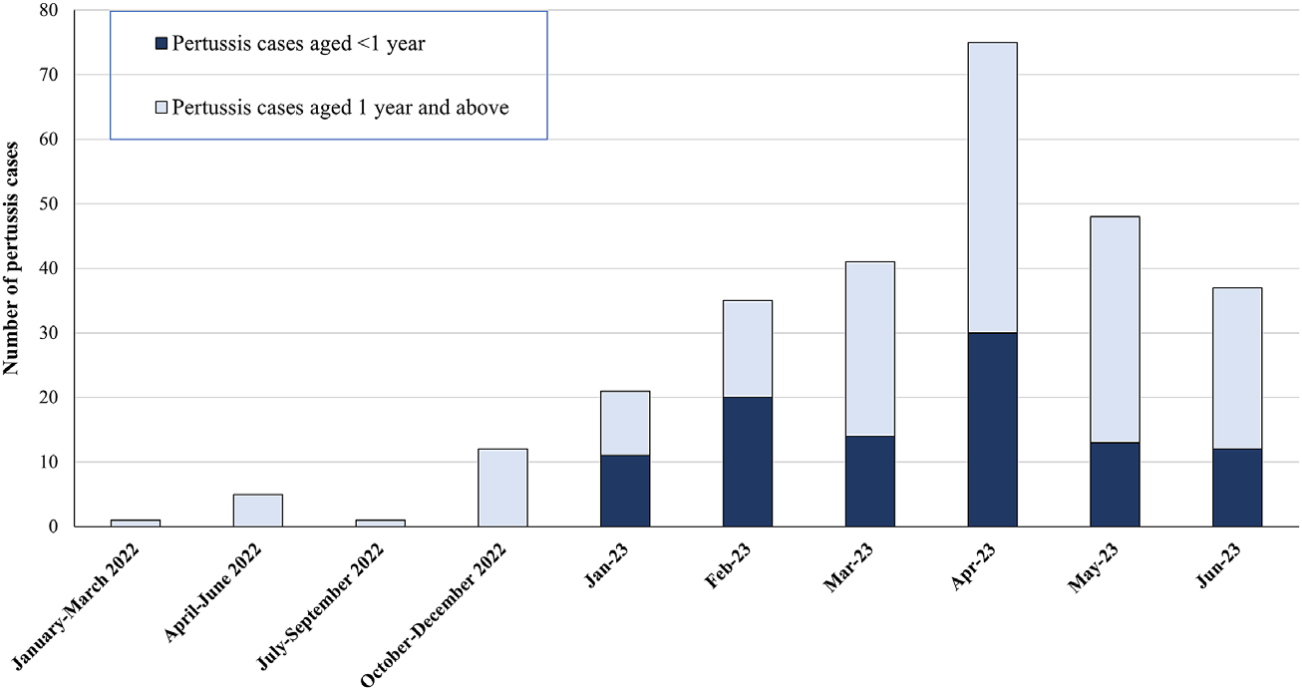
The epidemiological curve of notified pertussis cases, Jerusalem district, 2022 (by quartiles) and January–June 2023 (by month), with the fractions of cases in infants under 1 year and persons aged 1 year and above of all reported pertussis cases.

Figure 1 shows the epidemiological curve of the notified pertussis cases in Jerusalem district in 2022 (by quartiles) and during January–June 2023 (by month), with the fractions of cases in infants under 1 year of age and persons aged 1 year and above of all reported pertussis cases. In 2022, no pertussis cases were reported in infants in Jerusalem district. In 2023, the fraction of infants of all the reported pertussis cases in the district was 55.4% during January–February 2023 and then declined to 37.9% of cases during March–April 2023 and to 29.4% of cases during May–June 2023 (Mantel–Haenszel chi square for linear trend = 9.1, p-value = 0.003).

**Figure 2.**
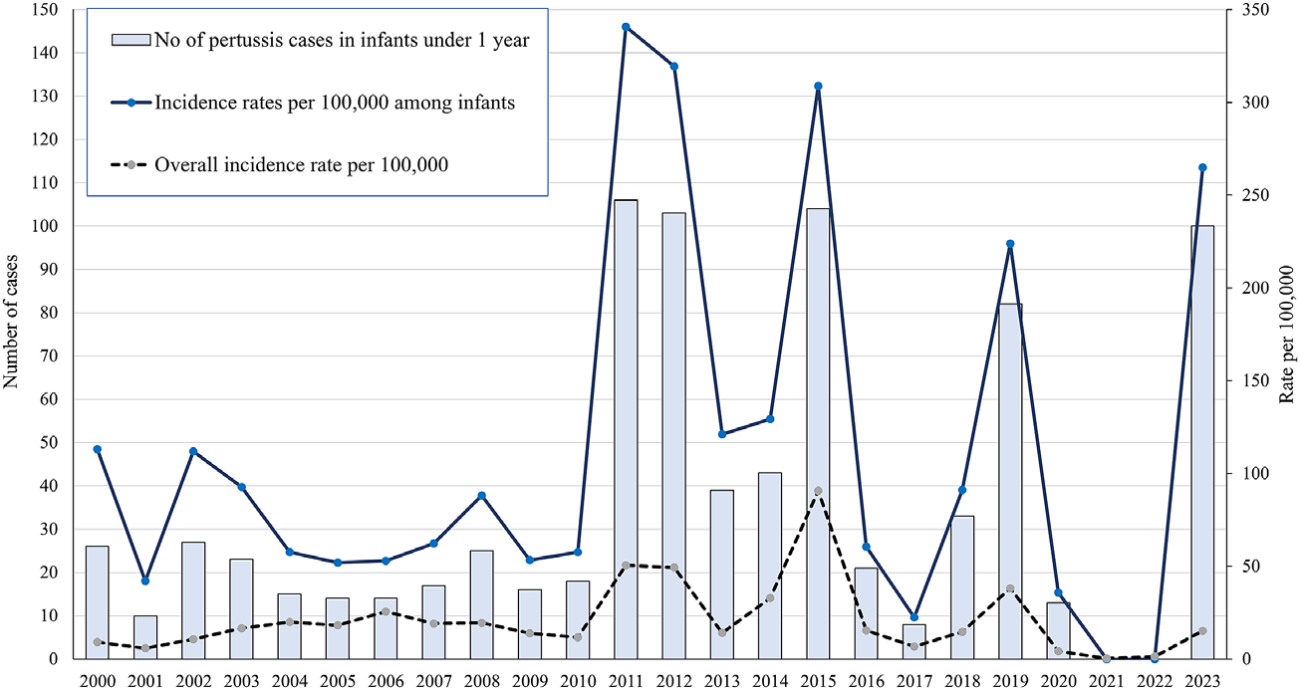
The annual number of reported pertussis cases in infants under 1 year in Jerusalem district in 2000–2023, the age-specific annual incidence rate of pertussis in infants under 1 year (per 10,000) and the district’s overall annual pertussis incidence rate (per 10,000 population).

Figure 2 presents the annual number of reported pertussis cases in infants under 1 year of age in Jerusalem district during the years 2000–2023, the age-specific annual incidence rate of pertussis in infants under 1 year of age (per 10,000), and the district’s overall annual pertussis incidence rate (per 10,000 population). Notably, there are considerable fluctuations in pertussis incidence rates between ‘endemic’ and ‘epidemic’ years. The 2023 pertussis outbreak has already reached, in the first six months of 2023, the extent of annual number of pertussis cases and incidence rates (in infants and overall) reported during previous pertussis outbreaks observed in the years 2011–2012, 2015 and 2019.

## The intervention

With the rise in pertussis notifications in January 2023, Jerusalem district health office issued an alert to all the health organisations (hospitals and health funds) to increase awareness, reporting on cases and promoting vaccinations. As the current outbreak emerged mainly among young children in under-vaccinated Jewish ultra-orthodox communities, the planning and application of control measures involved referring to the unique sociocultural characteristics of these communities. All the providers of preventive health services in Jerusalem district were continuously notified on the epidemiological data of the pertussis outbreak, as well as consulted about the coordination of the public health interventions.

The interventions included recruiting social marketing experts who are familiar with the communities and launching of a campaign for providing information (on the severity and consequences of pertussis, especially in young infants, and the importance of prevention) to the public in a culture-adapted manner. The target groups for intervention aiming to increase vaccination coverage in Jerusalem district were defined as 1. unvaccinated (‘zero doses’) and under-vaccinated infants and toddlers, and 2. pregnant women in the weeks 27–36 of pregnancy. According to the Ministry of Health Guidelines and aiming to rapidly protect the young infants, well-baby clinics in the district provide the first dose of pertussis-containing vaccine at the age of 6 weeks with the second and third vaccine doses provided at least 4 weeks apart [7, 12]. The families with unvaccinated children are repeatedly encouraged to approach the community well-baby clinics for catch-up vaccinations without the need to schedule an appointment.

The healthcare providers operate a system of reminders to pregnant women to come for the third-trimester pertussis vaccinations. The district’s primary care teams (physicians and nurses) received educational sessions (mainly through professional webinars) on pertussis and its complications and how to convey the message of the importance of vaccinations to their patients, with illustrations of frequent questions and answers.

A complex process of data linking is currently in progress to enable identification of children without documentation of registration at any of the preventive health service providers in the district (and hence unvaccinated). A dedicated call centre will approach the parents of the unvaccinated children telephonically and encourage them to bring the child for vaccinations as soon as possible. The process is still under development.

## Discussion

Pertussis outbreaks have emerged in various locations in the world during 2023, shortly after the global COVID-19 pandemic [11]. Pertussis resurgence and infection transmission are associated with underutilisation of pertussis vaccinations and waning immunity, leading to decreased herd immunity [3]. Implementation of comprehensive, sustainable, and updated pertussis vaccination strategies is essential, aiming to prevent and contain future outbreaks, as well as protection of risk groups, especially young infants [3]. The two main components necessary for promoting better pertussis control are improving vaccination strategies with the currently available vaccines and development of novel highly immunogenic and efficacious pertussis vaccines [6].

The fundamental preventive measure for successful control and elimination of vaccine-preventable diseases (including pertussis) is achieving sustained high vaccination coverage [13]. The Global Vaccine Action Plan goals were to achieve vaccination coverage of at least 90% with three doses of diphtheria–tetanus toxoid–pertussis-containing vaccine (DTP) by the year 2015 and at least 90% with all vaccines in the national programmes by the year 2020 [14]. According to the United Nations Children’s Fund (UNICEF) recent report, ‘The State of the World’s Children 2023’, the COVID-19 pandemic has devastatingly affected childhood vaccination coverage globally [15]. The vaccination coverage rates of DTP3 among 1-year-old children worldwide decreased from 86% in 2019 to 81% in 2021, with about 25 million children missing out on vaccination, 6 million more than that in 2019 and the highest number reported since 2009 [15]. In response, coordinated recovery plans are applied by international health organisations and agencies to restore the previous achievements of the Global Vaccine Action Plan in children.

In Israel, according to the vaccination report issued by the Ministry of Health in June 2023, the overall national vaccination coverage rates for the fourth dose of the diphtheria–tetanus–acellular pertussis (DTaP), DTaP4 vaccine for children (scheduled at the age of 12 months), declined from 96.8% in 2017 to 91% in 2022 [16]. Among children in Jerusalem district, the overall vaccination coverage rate for the DTaP4 vaccine decreased considerably from 93.7% in 2017 to 84.1% in 2022 [16]. We have previously described vaccination incompleteness and vaccination delays among children in Jerusalem district, prevailing predominantly in children in the Jewish ultra-orthodox communities, and associated with the emergence of recurrent vaccine-preventable diseases outbreaks [17]. These communities have also been the epicentre of previous measles, mumps, and pertussis outbreaks in Jerusalem district [17]. The recent event of the circulating vaccine-derived poliovirus type 3 (VDPV 3) in Jerusalem in 2022, with a clinical case of acute flaccid paralysis in a 3.8-year-old unvaccinated child and multiple positive environmental samples for VDPV 3, also occurred in these communities [18]. Large households and overcrowding characterise these communities. In our study, pertussis cases in infants had a high birth order, previously associated with delayed childhood vaccinations [17].

The highest disease burden of pertussis is repeatedly documented in infants under 1 year of age, specifically during the early months of life [1, 2, 5, 6, 19]. In a systematic review [19], a wide range of pertussis incidence rates were reported in infants younger than 2–3 months of age, some exceeding 1,000 per 100,000 population in several countries during outbreaks, and virtually all pertussis deaths occurred in this age group. Our data demonstrate that young infants present the highest burden of infection (with pertussis incidence rates in infants under 1 year of age, in Jerusalem district, reaching 300 per 100,000 population, during years of outbreaks) and of pertussis-related hospitalisations. In the last decade, many countries (including Israel) have introduced recommendations for the administration of pertussis vaccines during pregnancy, aiming to protect very young infants through the transfer of maternal antibodies [1, 6, 7, 19]. The pertussis vaccination strategies in pregnancy have been reported as being highly effective against pertussis infection and pertussis disease-related hospitalisations among infants under 3 months [1]. However, the compliance with maternal pertussis vaccinations in pregnancy varies widely between population groups, and in our group, the compliance (16%, as reported by the mothers in the epidemiological investigations) was inadequate. Infants’ protection is also achieved by timely administration of pertussis vaccinations, primary series and boosters [1, 6, 7, 19]. Yet, in our group, most eligible infants (85%) were unvaccinated and not registered in any clinic of the preventive health service providers in the district, despite the fact that these services are offered free of charge. Considerable efforts focused on the hard-to-vaccinate population groups are made by a coalition of the health organisations in Jerusalem district to promote maternal vaccinations, infants’ early registration to the free preventive services, and receipt of childhood vaccinations [17].

Our study has some limitations. It is a descriptive observational study of a pertussis outbreak in a defined district (Jerusalem) with most cases reported in unvaccinated children residing in Jewish ultra-orthodox communities. Most cases were young with a median age of 1.5 years and a notably high birth order indicating large households. Hence, generalisation of the findings might be difficult. The characteristics of the affected communities, household size and living conditions, that affect pertussis transmission may differ between population groups and settings. Also, laboratory confirmation of pertussis was performed by PCR tests. Possibly, milder clinical cases were underdiagnosed and not referred to PCR tests. Considering the characteristics of the involved communities and the likelihood of transmission, milder cases might have been missed. The vaccination status of children was verified via the national immunisation registry [8], while in persons aged 19 and above (7.8%), the information was based on the cases’ epidemiological investigations. Presumably, self-reporting might have caused bias in the adult group.

In conclusion, despite the availability of effective pertussis vaccines for over six decades, pertussis control is still inadequate, and so, *Bordetella pertussis* is circulating in many countries. The management of pertussis resurgence, particularly during the COVID-19 pandemic and beyond, necessitates a robust public health infrastructure. Improving and sustaining pertussis vaccination coverage in infants and young children, globally and locally, as well as provision of maternal vaccinations, are essential to preventing the high pertussis disease burden in young infants (pertussis-related hospitalisations and mortality). In hard-to-vaccinate communities, long-term programmes and constant investments are required to ensure prevention of pertussis outbreaks.
